# Molecular diversity in fusidic acid–resistant Methicillin Susceptible *Staphylococcus*  *aureus*

**DOI:** 10.1093/jacamr/dlae154

**Published:** 2024-10-05

**Authors:** Dalida Bivona, Emanuele Nicitra, Carmelo Bonomo, Maddalena Calvo, Giuseppe Migliorisi, Marianna Perez, Grete Francesca Privitera, Nicolò Musso, Stefania Stefani, Dafne Bongiorno

**Affiliations:** Department of Biomedical and Biotechnological Sciences, Medical Molecular Microbiology and Antibiotic Resistance Laboratory (MMARLab), University of Catania, 95123 Catania, Italy; Department of Biomedical and Biotechnological Sciences, Medical Molecular Microbiology and Antibiotic Resistance Laboratory (MMARLab), University of Catania, 95123 Catania, Italy; Department of Biomedical and Biotechnological Sciences, Medical Molecular Microbiology and Antibiotic Resistance Laboratory (MMARLab), University of Catania, 95123 Catania, Italy; U.O.C. Laboratory Analysis Unit, A.O.U. ‘Policlinico-San Marco’, Via S. Sofia 78, 95123 Catania, Italy; U.O.C. Laboratory Analysis Unit, A.O. ‘G.F. Ingrassia’, Corso Calatafimi 1002, 90131 Palermo, Italy; U.O.C. Laboratory Analysis Unit, A.O.U. ‘Policlinico-San Marco’, Via S. Sofia 78, 95123 Catania, Italy; Department of Clinical and Experimental Medicine, University of Catania, 95123 Catania, Italy; Department of Biomedical and Biotechnological Sciences, Biochemistry Section, University of Catania, 95123 Catania, Italy; Department of Biomedical and Biotechnological Sciences, Medical Molecular Microbiology and Antibiotic Resistance Laboratory (MMARLab), University of Catania, 95123 Catania, Italy; U.O.C. Laboratory Analysis Unit, A.O.U. ‘Policlinico-San Marco’, Via S. Sofia 78, 95123 Catania, Italy; Department of Biomedical and Biotechnological Sciences, Medical Molecular Microbiology and Antibiotic Resistance Laboratory (MMARLab), University of Catania, 95123 Catania, Italy

## Abstract

**Objectives:**

The recent emergence of fusidic acid (FA)–resistant *Staphylococcus aureus* has underscored the importance of active surveillance in isolating these strains. The molecular basis of fusidic acid resistance and the carriage of virulence factors in four borderline oxacillin-resistant *Staphylococcus aureus* (BORSA) clinical strains was assessed through phenotypical and genotypical methods.

**Methods:**

All *S. aureus* clinical strains were obtained from various hospital units in Sicily*. In vitro* antibiotic susceptibility testing was conducted. WGS was performed using the Illumina MiSeq Platform, and data analysis was carried out to determine ST, resistome and virulome profiles.

**Results:**

Genotypic characterization revealed that the strains belong to four STs: ST630, ST8, ST15, and ST1. FA resistance was associated with mutations in the *fusA* gene or *fusB* and *fusC* genes. Additionally, one case exhibited resistance to mupirocin, related to the presence of the *mupA* gene. Borderline MIC values were observed for cefoxitin in three out of four cases, leading to their categorization as BORSA. Virulence gene content was complex and diversified, with one testing positive for the *lukS/F* genes, coding for PVL toxin.

**Conclusions:**

Resistance to FA is multifactorial, involving point mutations in chromosomal genes or association with mobile genetic elements. Monitoring the resistance to these antibiotics might help to manage and eradicate mupirocin- and FA-resistant *S. aureus* strains, which are also known to be important carriers of virulence determinants.

## Introduction

Recent concern about the appearance of fusidic acid (FA)–resistant *Staphylococcus aureus* encourages active surveillance to identify and characterize these strains.^1^ Indeed, recently Vendrik *et al.*^2^ reported a community outbreak of impetigo caused by MRSA, with additional resistance to FA—a troubling phenomenon in a country with low antibiotic resistance prevalence among young children. This type of outbreak can occur not only among MRSA but also in MSSA.^1^ FA is a narrow-spectrum antibiotic that acts by inhibiting the elongation factor G at the ribosome level and blocking protein synthesis.^3^ FA usage in clinical practice mainly consists in the topical treatment of cutaneous and soft tissue infections. In some cases, such as chronic bone and joint infections, it is also employed in systemic therapy in combination with other antibacterial agents effective against *S. aureus* infections.^4^

FA-resistant *S. aureus* strains are globally widespread and have usually been reported as susceptible to several antibiotic classes like β-lactams, macrolides and aminoglycosides. This distinctive characteristic may arise from the inherent FA chemical structure.^1^

Chromosomal mutations in the *fusA* gene encoding the elongation factor EF-G, in *fusE* and in genes associated with EG-F functionality (*fusB*-*fusC*-*fusD*) have been described as relevant factors in FA resistance. FA-resistant *S. aureus* represents a threat for different reasons: (i) above all, a possible decreased bacterial cell wall or membrane permeability could promote plasmid-mediated FA resistance, allowing the spread of these resistance determinants to other permissive strains or species;^5,6^ and (ii) in addition to the antibiotic resistance genes, mobile genetic elements might carry various genes for virulence factors including toxins, enzymes and adhesins. This makes these strains key players in disseminating virulence factors among tissues and other bacteria.^7^

### Objectives

Four isolates of FA-resistant methicillin-susceptible *S. aureus* (FAR-MSSA), collected from paediatric patients in three out of four cases and admitted to different Sicilian hospitals, came to our attention for a complete phenotypical and molecular characterization. This approach clarifies the molecular basis beyond the FA resistance mechanism and promotes a determination of the virulence factors because their dissemination among *S. aureus* strains could be involved in the worsening development of clinically detected disease.

## Methods

Three out of four *S. aureus* clinical strains were collected in two hospital units in Catania, and the fourth in Palermo, Italy, during 2023. Antibiotic susceptibility via E-test was conducted according to CLSI guidelines,^8^ and categorized by EUCAST interpretation criteria.^9^

Several antibiotics were tested: oxacillin, penicillin G, cefoxitin, ceftobiprole, ceftaroline, tetracycline, tigecycline, ciprofloxacin, levofloxacin, gentamicin, oritavancin, teicoplanin, vancomycin, quinupristin/dalfopristin, erythromycin, clindamycin, linezolid, daptomycin, chloramphenicol, rifampicin, trimethoprim/sulfamethoxazole, fosfomycin, fusidic acid and mupirocin.

The β-lactamase production was assessed by synergic test as previously described.^10^ WGS was performed using the Illumina MiSeq Platform. Data were analysed using QIAGEN CLC Genomics Workbench software, in order to establish ST, resistome and virulome profiles. For analysis of the *agr* locus, staphylococcal chromosomal cassette *mec* (SCC*mec*), spa-type and plasmid the following software was used: bactopia using its ‘bactopia tools’ such as AgrVATE, staphopia-sccmec,^11^  *spa*Typer and plasmidfinder.^12^ The raw fastq files were *de novo* assembled using Unicycler.^13^ FA resistance determined by mutations in the *fusA* gene was assessed by comparing genomes with the deposited sequence in the Comprehensive Antibiotic Resistance Database (CARD) (>gb|BX571856.1|+|599703-601784|). FA resistance associated with the *fusB* gene, carried by p*UB*101 plasmid, was determined using WP_000855537.1 as reference sequence provided by the NCBI. The *eta* gene was analysed using BLAST, comparing our sequences with NCBI reference sequence NC_009641.1 (https://www.ncbi.nlm.nih.gov/nuccore/NC_009641.1?from=1186112&to=1187059).

## Results

Antibiotic molecules belonging to different classes (cephalosporins, tetracyclines, fluoroquinolones, aminoglycosides, glyco-lipopetides, macrolides, streptogramins, lincosamides and oxazolidinones) were tested and susceptibility was reported for all strains; MIC values and EUCAST breakpoints were reported for the most important molecules involved in resistance to β-lactam antibiotics, mupirocin and FA (Table 1).

**Table 1.** Principal clinical, phenotypical and genotypical characteristics of the studied strains

Sample	Case 1	Case 2	Case 3	Case 4
Origin	Paediatric	Adult	Paediatric	Paediatric
Source	Skin	Skin	Skin	Blood culture
MLST/CC/spa-type	ST630/CC8/t377	ST8/CC8/t008	ST15/CC15/t084	ST1/CC1/t127
**Antibiotic susceptibility test—MIC (mg/L)**				
Penicillin G S ≤ 0.125; R > 0.125	2	0.047	1	2
Oxacillin	0.5	0.06	0.125	0.5
Cefoxitin S ≤ 4 S; R > 4	4	1	4	4
Ceftobiprole S ≤ 2; R > 2	0.5	0.38	0.38	0.5
Ceftaroline S ≤ 1; R > 2	0.25	0.25	0.5	0.38
Fusidic acid S ≤ 1; R > 1	8	8	16	32
Mupirocin	0.19	0.38	0.38	>1024
Induced resistance to macrolides	NEG	NEG	POS	NEG
**β-Lactam resistance gene^a^**				
*blaZ*	POS	NEG	POS	POS
**Genes related to fusidic acid resistance^b^**				
*fusA*	NEG	POS	POS	NEG
*fusB*	POS	NEG	NEG	NEG
*fusC*	NEG	NEG	NEG	POS
**Virulence genes^c^**				
*PVL*	NEG	NEG	NEG	POS
*eta*	POS	POS	POS	POS
*eta* mutations	E127K	E127K	K305N	C168S, L222I

NEG, negative; POS, positive; R, resistant; S, susceptible.

^a^ *blaZ,* β-lactamase.

^b^ *fusA*, gene associated with fusidic acid resistance encoding for translation factor GTPase family; *fusB*, gene associated with fusidic acid resistance encoding for elongation factor G-binding protein; *fusC*, gene associated with fusidic acid resistance encoding for elongation factor G-binding protein.

^c^ *eta*, exfoliative toxin A; *PVL*, Panton–Valentine leucocidin.

Although the reported MIC values for cefoxitin were in a range of susceptibility, high MIC values, equal to 4 mg/L, were reported for cases 1, 3 and 4 (Table 1). All the isolates were strong β-lactamase producers, as demonstrated by a synergism test between cefoxitin and ampicillin/sulbactam, showing a decreased cefoxitin MIC value due to the effect of β-lactamase inhibitor. Based on these results, these cases were categorized as borderline oxacillin-resistant *Staphylococcus aureus* (BORSA).

Regarding the quality of the genotypical downstream analysis, the average number of reads of the four genomes was equal to 4 144 518 with 221× coverage.

The strains belonged to four diverse ST/clonal complex–*spa*-types: ST630/CC8-t377 (case 1), ST8/CC8-t008 (case 2), ST15/CC15-t084 (case 3) and ST1/CC1-t127 (case 4).

The FA resistance was related to mutations in *fusA* in cases 2 and 3 (MIC values 8–16 mg/L), to *fusB* (8 mg/L) in case 1 and to *fusC* (32 mg/L) in case 4 (Table 1). In addition, a high level of mupirocin resistance, equal to >1024 mg/L, was registered for case 4. Analysing the case 4 genome, the presence of a plasmidic gene called *mupA*, encoding an isoleucyl-RNA synthetase, could account for the resistance. A comprehensive analysis of the genetic characteristics of the isolates is presented in Table S1 (available as Supplementary data at *JAC-AMR* Online), revealing the detection of multiple virulence factors across all isolates.

The ST630-CC8-MSSA (case 1) was isolated from a newborn with staphylococcal scalded skin syndrome (SSSS) and carried the highly characteristic SSSS *eta* gene,^14^ encoding exfoliative toxins. The genes *vWbp* and *ccrB* were present. This isolate was also found to carry *fusB*, inserted into the pBU101 plasmid, together with *blaZ* associated with the plasmid rep16.

The ST8-CC8-MSSA (case 2) was recovered from an adult patient with thoracic skin lesions, and the isolate also contained the leucocidin genes, *lukED*, as well as other adhesin genes such as *sdrE*, *sdrD* and *vWbp* which would support its implication in staphylococcal soft skin infections (SSSIs).^15^

The ST15-CC15-MSSA (case 3) was isolated from a paediatric patient with SSSI, showing inducible macrolide resistance confirmed by the D test. Here again, the *blaZ* gene was associated with the same rep16 plasmid.

The ST1-CC1-MSSA (case 4) was associated with a bloodstream infection in a paediatric patient suffering from chronic osteomyelitis. Thus, this strain uniquely expressed genes of the microbial surface components recognizing adhesive matrix molecules (MSCRAMM) family (*clfA/B*, *sdrC*, *fnbA/B*, *cna*),^16^ the coagulase factor Coa, and also *lukS/F* genes coding the PVL toxin, which may contribute to osteomyelitis.^17^

### Discussion

Overall, the FAR-MSSA strains isolated in our geographical area were categorized as BORSA and showed different genetic backgrounds, with a plethora of virulence genes. In the literature the isolation of FAR strains has been reported mainly in MSSA particularly in Europe. A recent systematic review of the literature reports an increment of Fusidic Resistant *Staphylococcus aureus* in different areas of the globe, i.e. Africa (6.8%), Asia (5.6%), Oceania (5%) and America (4.3%) with respect to Europe (1.9%). Globally, it has been reported as gradually increasing from 4% before 2000 to 5.2% in 2010–2020.^1^

The spread of community-associated FAR-MSSA in children and adolescents was recently related to an ST121 clone harbouring exfoliative toxin in Greece.^18^ In a recent study, conducted in the United Arab Emirates, eight FAR-MSSA were described associated with *fusC*, in strains belonging to CC1.^19^ Despite the low number of cases reported until now, strict monitoring of FA-resistant strains, even those susceptible to most antibiotic molecules, is crucial in both community and healthcare settings. Monitoring resistance to these antibiotics may help manage and eradicate *S. aureus* mupirocin- and FA-resistant strains, as well as counteract the spread of important virulence determinants. *S. aureus* strains exhibiting a similar profile may be associated with outbreaks; thus, an active surveillance and cooperation between clinical and molecular microbiology laboratories becomes pivotal in managing dissemination and preventing diseases attributed to these pathogens.

## Supplementary Material

dlae154_Supplementary_Data

## Supplementary data

Table S1 is available as Supplementary data at *JAC-AMR* Online.
