# Supplementary material for: Molecular diversity in fusidic acid–resistant Methicillin Susceptible Staphylococcus aureus
Source: JAC Antimicrob Resist. 2024 Oct 5;6(5):dlae154. doi: 10.1093/jacamr/dlae154 (PMC11452824; doi:10.1093/jacamr/dlae154)
Supplement: dlae154_Supplementary_Data [file dlae154_supplementary_data.docx]

Supplementary matherial

Table S1. Molecular characterization of studied strains.

| **Sample** | **Strain assignment** | **Accesory gene regulator (agr)** | **Capsular type** | **Resistance genes** | **Virulence genes** | **Plasmids (Accession number)** |
| --- | --- | --- | --- | --- | --- | --- |
| Case 1 | ST630-CC8-MSSA-[*fus+ccrB*] | *agr I* | *cap 5* | ***fusB****, fosB, tet38, mepA, mepR, LmrS, mgrA, arlR, arlS, norA,* ***blaZ*** | *hla, hlb, hld, hlgA, hlgC, hlgB, aur, map, ebp, geh, lip, sspB, sspC, sak, esxA, esxB, esaA, esa, esaC, essA, essB, essC, esaC, vWbp, eta* | rep16 (AY917098); rep21 (NC007790); rep7c (BX571857) |
| Case 2 | ST8-CC8-MSSA-[fus] | *agr I* | *cap 5* | ***fusA****, fosB, tet38, mepA, mepR, LmrS, arlS, mgrA, arlR, norA* | *hla, hlb, hld, hlgA, hlgC, hlgB, aur, chp, map, ebp, geh, lip, scn, sdrD, sdrE, sspB, sspC, sak, esxA, esxB, esaA,esaB, esaC, essA, essB, essC, sspA, vWbp, LukD, LukE, eta* | rep7c (BX571857) |
| Case 3 | ST15-CC15-MSSA-[fus] | *agr II* | *cap 8* | ***fusA****, fosB, tet38, mepA, mepR, LmrS, mgrA, arlR,* ***ermA****, norA,* ***blaZ*** | *hla, hlb, hld, hlgA, hlgC, hlgB, aur, chp, clfA, clfB, lip, geh, scn, sspB, sspC, esxA, esaA, essA, esaB, essB, ebp, eta* | rep16 (CP002115) |
| Case 4 | ST1-CC1-MSSA-[fus] | *agr III* | *cap 8* | ***fusC****, tet38, mepA, mepR, LmrS, mgrA, arlR, arlS, norA,* ***blaZ****, mupA* | *hla, hlb, hld, hlgA, hlgB, hlgC, adsA, aur, chp, clfB, clfA, ebp, fnbA, fnbB, hysA, lip, geh, scn, sdrC, sdrD, sdrE, sea, seh, sbi, spa, sspB, sspC, coa, sak, esxB, esaA, esaB, esaC, essA, essB, essC, esxA, sspA, vWbp, lukS-PV, lukF-PV, eta* | rep16 (BX571858); rep7c (BX571857) |

***agr****,* accessory gene regulator; ***ccr****,* cassette chromosome recombinase gene; ***blaZ****,* beta-lactamase; ***fusA****,* gene associated with fusidic acid resistance encoding for translation factor GTPase family; ***fusB****,* gene associated with fusidic acid resistance encoding for elongation factor G-binding protein; ***fusC****,* gene associated with fusidic acid resistance encoding for elongation factor G-binding protein; ***fosB*** gene that leads to the resistance of fosfomycin; ***tet38,*** *tetracycline efflux pump;* ***mepA****,* gene for efflux pump protein; ***mepR***, gene for upstream repressor of MepA; ***lmrS***, secondary active transporters; ***mgrA****,* regulator for norA, norB, and tet38; ***arlR***, response regulator for norA; ***arlS****,* protein histidine kinase for ArlR; ***ermA***, ribosomal RNA methyltransferase; ***ermC***, ribosomal RNA methyltransferase; ***norA***, multidrug efflux pump; mupA, antibiotic-resistant isoleucyl-tRNA synthetase ***hla***, alpha-hemolysin; beta-hemolysin; ***hld***, delta-lysin; gamma-hemolysin A; ***hlgB***, gamma-hemolysin B; ***hlgC***; gamma-hemolysin C; ***hly***, alpha-hemolysin; ***adsA***, adeosine synthase A; **aur**, aureolysin; ***chp***, chemotaxis inhibitory protein; ***cap****, capsular gene;* ***clfA***, clumping factor A; **clfB**, clumping factor B; ***ebp***, elastin-binding protein; ***fnbA***, fibronectin-binding protein A; **fnbB**, fibronectin-binding protein B; ***hysA***, hyaluronate lyase A; ***scn***, staphylococcal complememnt inhibitor; ***sdrC***, serine-aspartate repeat-containing protein C; **sdrD**, serine-aspartate repeat-containing protein D; ***sdrE***, serine-aspartate repeat-containing protein E; ***sea***, staphylococcal enterotoxin A; **seh**, staphylococcal enterotoxin H; ***sbi***, second immunoglobulin-binding protein; ***spa***, staphylococcal protein A; ***sspA***, staphylococcal serin-protease A; ***sspB***, staphylococcal serin-protease B; ***sspC***, staphylococcal serin-protease C; ***coa***, coagulase gene; ***sak***, staphylokinase; ***esxA***, staphylococcal protein secretion system A; ***esxB***, staphylococcal protein secretion system B; ***esaA***, staphylococcal secretion system component A; ***esaB***, staphylococcal secretion system component B; ***esaC***, staphylococcal secretion system component C; ***essA***, staphylococcal secretion machinary protein A; ***essB***, staphylococcal secretion machinary protein B; ***essC***, staphylococcal secretion machinary protein C; ***vWbp***, von Willebrand factor-binding protein; ***eta***, exfoliative toxin A; ***map***, MHC class II analog protein; ***ebp***, elastin binding protein; **lukS*/F*** PV, Panton Valentine leukocidin encoding factors; ***lukD***, leuko-toxin encoding factor D; ***lukE***, leuko-toxin encoding factor E; **MSSA**, methicillin-susceptible *Staphylococcus aureus;* **CC,** Clonal Complex
